# Late complications of radiosurgery for cerebral arteriovenous malformations: report of 5 cases of chronic encapsulated intracerebral hematomas and review of the literature

**DOI:** 10.1186/s13014-020-01616-1

**Published:** 2020-07-22

**Authors:** Stephanos Finitsis, Valerie Bernier, Isabelle Buccheit, Olivier Klein, Serge Bracard, Francois Zhu, Guillaume Gauchotte, René Anxionnat

**Affiliations:** 1 Aristotle University of Thessaloniki, Ahepa Hospital, Kyriakidi 1, 54621 Thessaoniki, Greece; 2 Centre Alexis Vautrin, Institut de Cancérologie de Lorraine, 6 avenue de Bourgogne CS 30519, 54519 Vandoeuvre-lès-Nancy Cedex, France; 3 Hôpital d’Enfants, CHU de Nancy – Hôpitaux de Brabois, Rue du Morvan, 54511 Vandoeuvre-lès-Nancy Cedex, France; 4 Service de Neuroradiologie Diagnostique et Thérapeutique, Hôpital Universitaire de Nancy, 29 avenue du maréchal de Lattre de Tassigny CO 60034, 54035 Nancy, France; 5 Département de Biopathologie - Anatomie et Cytologie Pathologiques, CHRU de Nancy - CHRU/ICL - bâtiment BBB, Rue du Morvan, 54511 Vandoeuvre-lès-Nancy, France

## Abstract

**Background:**

Chronic encapsulated intracerebral hematomas (CEIHs) are a rare, late complication of radiosurgery for intracranial AVM. We present 5 cases treated mostly by surgical excision and review the literature.

**Methods:**

Patients (age 39, 42, 36, 31, 62) presented with headache, paresthesia, hemiparesis or were asymptomatic. CEIHs presented 10 to 13 years (median 12 years) post radiosurgery. Three patients had demonstrated early radiation induced changes post radiosurgery. Angiographic cure, assessed with DSA, was present in all cases except 1 case with a small nidus remnant. MRI demonstrated mixed lesions with a solid enhancing part, organized hematoma and extensive surrounding edema while three cases had also a cystic component.

**Results:**

Excision of the CEIHs with complete or partial removal of the capsule was performed in 4 patients and resulted in marked clinical improvement. One patient was managed conservatively with administration of steroids as surgery was judged excessively hazardous with eventual stabilization of his symptoms.

**Conclusions:**

CEIHs are rare, late complications of radiosurgery for cranial AVM. They may be asymptomatic or provoke symptoms and may be preceded by early radiation induced changes. Complete removal of CEIHS is an effective treatment. Because of the long latency period of CEIHs, patients who had radiosurgery for brain AVMs should be followed by MRI at least 10 years even after complete obliteration.

Stereotactic radiosurgery (SRS) has become an alternative or complementary treatment for brain arteriovenous malformations (AVMs) especially for lesions that are small (< 3 cm), large and complex, or located in eloquent areas [1, 2]. Delayed complications post bAVM SRS are rare, typically detected 5 or more years after SRS. They include cyst formation, de novo cavernoma formation and chronic encapsulated intracerebral hematoma (CEIH) [3–8]. They are distinct from radiation-induced changes (RICs) noted in the first 1 to 2 years after AVM SRS (areas of increased T2 signal) and radionecrosis [9–13]. Delayed complications can cause mass effect and, if symptomatic, may require surgical intervention [14].

We report 5 cases of CEIH that developed in patients with bAVMs that had been completely obliterated using SRS and review the literature.

## Materials and methods

The Neurointerventional Department of the University of Nancy, Nancy, France, is a tertiary center serving a region of 2,35 million inhabitants. We reviewed our medical records for the period 1997 to 2014 during which 288 patients were treated with radiosurgery for a brain arteriovenous malformation. We obtained the presentation, diagnosis, management and clinical outcomes of 5 cases of interest. Because of the retrospective nature of the study, permission from the ethics committee of our institution was not necessary. This research complies with the STROBE (Strengthening the Reporting of Observational studies in Epidemiology) reporting guidelines. We also performed a comprehensive literature search using Pubmed. The following key words were queried singly and in combination: arteriovenous malformation, brain, hematoma, radiosurgery. Our search resulted in case reports and cases series describing CEIH post bAVM SRS. In all cases that could be extracted (including ours and the cases in the referenced articles), we collected the clinical presentations, imaging findings, management and outcome.

### Illustrative cases

**Fig. 1:**
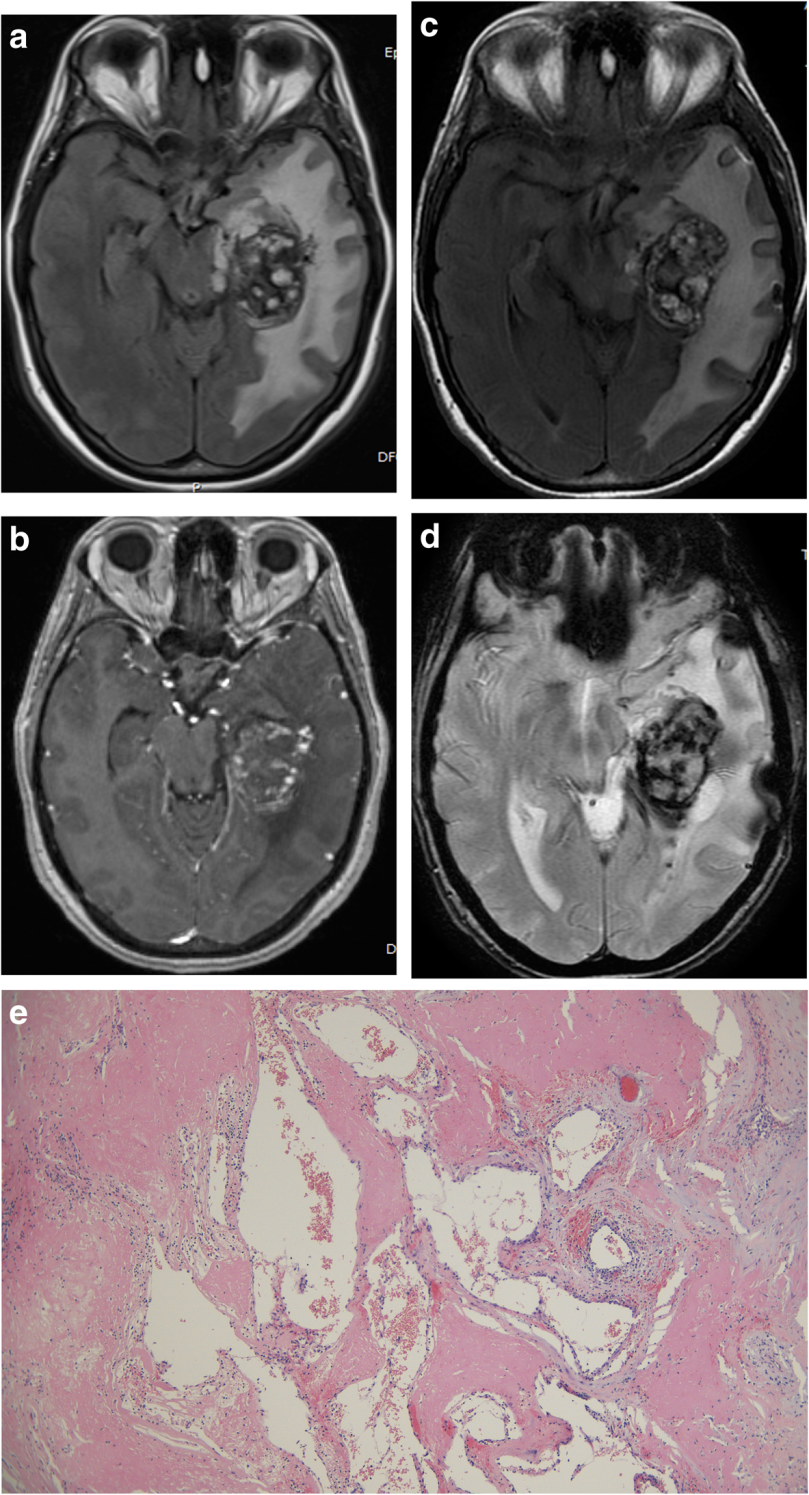
Case 1. A 27-year-old woman presented with hemorrhage due to a left temporal AVM. After partial treatment with embolization, 18 Grays were delivered to the margin of the lesion with a collimator of 20 mm. Twelve years post SRS the patient complained of chronic headache. **a** Axial Flair and (**b**) axial T1 contrast enhanced images showed a large heterogeneous well delineated cavernoma-like lesion. Surgery was initially recused because of the deep location and the mild symptoms of the patient. **b** Axial Flair, **c** axial T2* MRI images 2 years later showed lesion growth which was surgically removed. **d** Histology revealed an angiomatous pseudo-cavernous lesion containing dilated vascular lumen, fibrino-hemorraghic changes and fibrosis (hematoxylin, eosin and saffron, × 100)

**Fig. 2:**
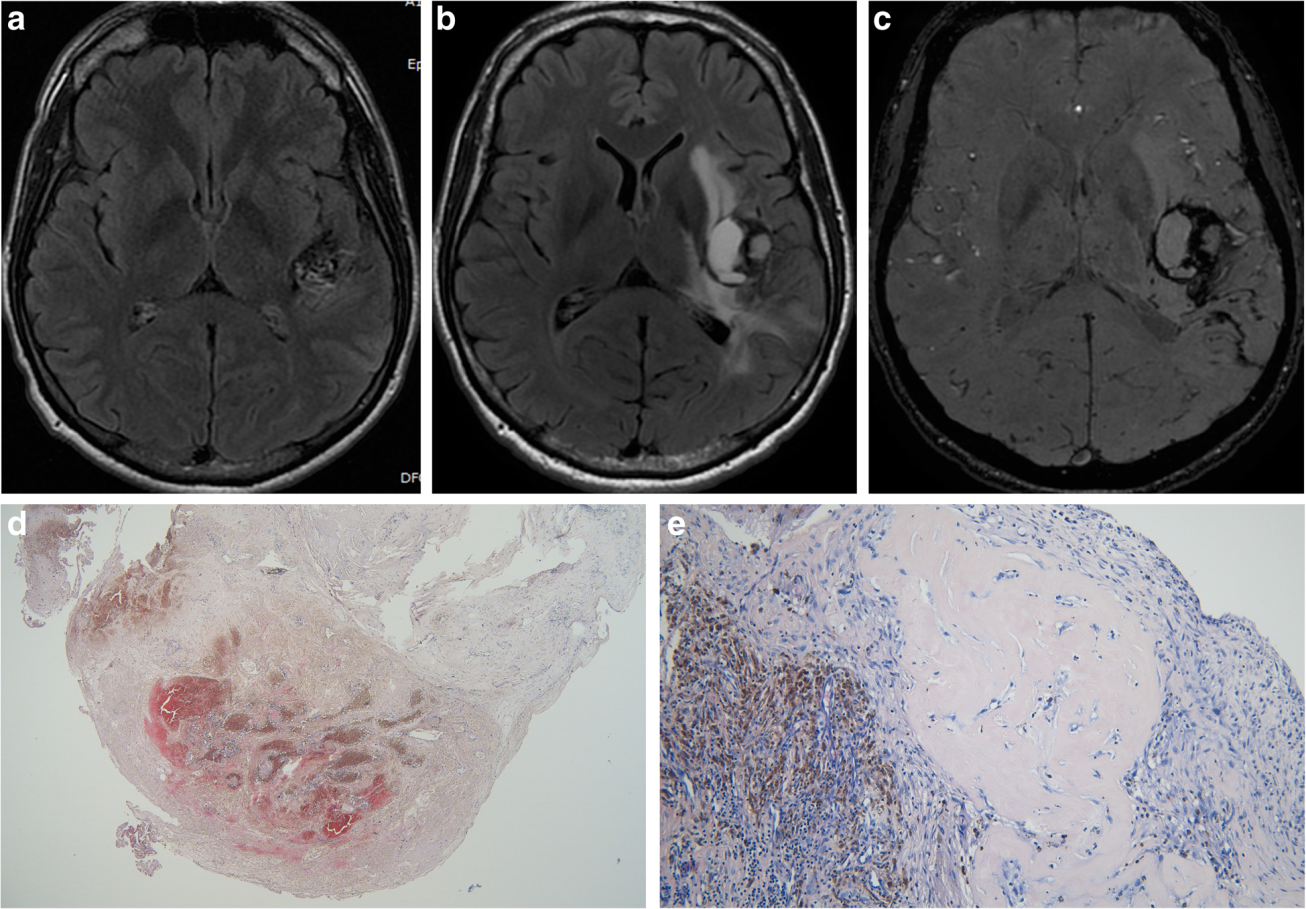
Case 4. A 21-year-old man presented with seizures related to a left temporal AVM. The lesion was partially treated with embolization. A nidal remnant was further treated with 18 Grays delivered at the periphery of a target volume of 5,3 ml. Two years post SRS an MRI showed mild radiation induced changes. Ten years post SRS, the patient presented with headache and paresthesia. **a** axial Flair at the time of SRS. Axial Flair (**b**) and (**c**) SWI 11 years later show a large cystic lesion with chronic hemorrhage and an enhancing nodular part with surrounding edema. The cyst was evacuated, and the walls partially removed. Histology showed (**d**) a well-circumscribed vascular lesion with extensive fibrosis (hematoxylin, eosin and saffron, × 40) and (**e**) post radiation hyalinization of small vessels (hematoxylin, eosin and saffron, × 400)

**Fig. 3:**
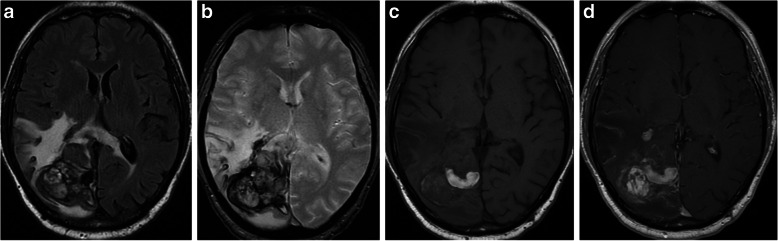
Case 5. A 49-year-old man presented with intracranial hemorrhage related to a right occipital AVM. The lesion was partially treated with embolization. A nidal remnant was further treated with SRS with a marginal dose of 18 Grays. Thirteen years post SRS, the patient presented hemiparesis. **a** Flair, (**b**) T2*, (**c**) T1 and d) T1 contrast enhanced MR images show a large heterogeneous cavernoma-like lesion with extensive edema. Surgery was judged as too risky and the lesion finally stabilized under steroid therapy

Three of the 5 patients were males. The mean patient age was 30,8 years (range 27 to 49 years). The presenting symptom was seizures in 3 cases and hemorrhage in 2 cases. AVMs were located in the temporal lobe in 2 cases, and the parietal, frontal and occipital lobes in the other cases. All lesions were partially embolized prior to radiosurgery. The marginal dose delivered was 18 Grays for all lesions. The mean irradiated nidus volume was 5,2 ml (range 3,8 to 9 ml). Four of five AVMs were angiographically obliterated post radiosurgery. Three cases demonstrated RICs following radiosurgery. The mean time of CEIH presentation was 10,2 years post radiosurgery (range 6 to 13 years). MRI studies showed hemorrhagic lesions with extensive peri-lesional edema (Fig. 1). A cyst coexisted with the hemorrhagic lesion in 3 cases (Fig. 2). Three cases were immediately treated with surgical excision of the hemorrhagic lesion and the cystic component. Two cases were treated conservatively with steroid administration. One of these cases showed interval growth and symptom worsening after 2 years and was eventually treated surgically while the other case remained clinically stable during a follow-up period of 8 years (Fig. 3). All surgically treated cases demonstrated typical histological features of CEIH and clinical improvement.

### Literature search

We found 32 cases of CEIH in case reports and case series. Reports with insufficient or no data were excluded. In total, 37 cases including our own 5 cases were analyzed (Table 1).

**Table 1:** Reported cases of CEIHs

Author	Age (years)	Sex	Initial Bleeding	Locat-ion	Embo-lization	Radio- surgery Source	Marginal Dose (Gy)^a^	RIC/ RN^b^	Cyst	Years post SRS	Symptoms	MR Imaging	AVM Complete Obliteration	Treatment	Outcome^**b**^
**1996 Kurita** [7]	23	M	+	IC	–	GKS	20	RN	–	2	HH, N/V	ns	–	Hematoma evacuation, Excision	Improvement
**2006 Maruyama** [15]	51	M	–	BG	–	GKS	22,5	–	–	6	HH	Hypo L	–	Excision	Improvement
**2008 Motegi** [8]	47	M	–	BG	–	Linac	25	–	+	7,5	HH	HR, Het L, Nod E	+	Excision	Improvement
**2008 Pan** [16]	10	M	ns	L	–	GKS twice	ns	–	–	5	HH, HP	Het L	+	Excision	Improvement
**2009 Takeuchi** [1]	15	F	+	BG	–	Linac	ns	–	+	7	HP	HR, Het L	+	Ommaya Reservoir	Improvement
**2010 Nakazimo** [17]	57	M	–	BG	–	GKS	22,5	–	+	5	AS	ns	–	Excision	Stable
**2010 Nakamizo** [17]	55	M	–	L	–	Linac	20	–	–	11	HP	HR, Hypo L	+	Excision	Improvement
**2010 Nakamizo** [17]	15	F	+	BG	+	GKS	18	RIC	–	3	HH, HP, Vis	HR, Hypo L	–	Steroids, Excision	Improvement
**2011 Lee** [18]	10	M	+	L	–	GKS twice	18^a^	–	–	5	HP, Vis	HR, Het L, Multi Nod E	+	Excision	Improvement
**2011 Takeuchi** [19]	49	M	–	BG	+	Linac	18	RN	+	4	SD, Memory	Nod E	+	Excision	Improvement
**2014 Watanabe** [20]	34	F	+	C	–	GKS twice	22^a^	RIC	+	13	HH, N/V, FP	HR, Hypo L, Nod E	+	Excision	Improvement
**2015 Park** [14]	30	F	–	L	–	GKS twice	28^a^	RIC	–	7	HP	Nod E	+	Steroids, Excision	Improvement
**2015 Park** [14]	36	F	–	L	–	GKS twice	30^a^	RIC	–	7	HP	HR, Het L	+	Steroids, Excision	Improvement
**2015 Park** [14]	16	M	–	L	+	GKS	25	RIC	–	3	HH	HR, Het L	+	Steroids, Excision	Improvement
**2015 Park** [14]	15	F	–	L	–	GKS three times	15^a^	RIC	–	2	HP	ns	+	Steroids, Excision	Improvement
**2015 Park** [14]	38	M	+	BG	–	GKS	25	–	+	12	HP	ns	+	Steroids, Partial Excision	Worse
**2015 Shuto** [21]	23	M	+	L	–	GKS	18	–	+	8,1	S	ns	+	FU, Excision	Improvement
**2015 Shuto** [21]	19	F	+	L	+	GKS	18	–	–	11,2	HH	Nod E	+	FU, Excision	Improvement
**2015 Shuto** [21]	33	M	–	L	+	GKS	18	–	+	4,5	HP, E	HR, Het L	+	FU, Excision	Improvement
**2015 Shuto** [21]	19	F	ns	ns	ns	GKS	28	–	+	10,3	ns	ns	–	FU, Excision	ns
**2015 Shuto** [21]	31	M	ns	ns	ns	GKS	25	–	+	5	ns	ns	+	FU, Excision	ns
**2015 Shuto** [21]	24	M	ns	ns	ns	GKS	20	–	+	1,1	ns	ns	–	FU, Excision	ns
**2015 Shuto** [21]	56	F	ns	ns	ns	GKS	20	–	+	12	ns	ns	+	FU, Excision	ns
**2015 Shuto** [21]	36	F	ns	ns	ns	GKS	18	–	+	7,9	ns	ns	+	FU, Excision	ns
**2015 Shuto** [21]	35	M	ns	ns	ns	GKS	18	–	+	7,1	ns	ns	+	FU, Excision	ns
**2015 Shuto** [21]	46	F	ns	ns	ns	GKS	20	–	+	6,2	ns	ns	+	FU, Excision	ns
**2015 Shuto** [21]	50	F	ns	ns	ns	GKS	20	–	+	10,1	ns	ns	+	Lost FU	Lost FU
**2015 Shuto** [21]	47	M	+	L	–	GKS	25	–	+	6,2	Vis	Hypo L	+	Lost FU	Lost FU
**2015 Shuto** [21]	17	F	–	L	–	GKS	18	–	+	3,2	HH	Nod E	+	Omaya Reservoir, Excision	Improvement
**2016 Takei** [22]	37	M	+	L	–	ns	ns	–	+	15	HH, N/V, AT	HR	+	Partial Excision	Improvement
**2019 D’Aliberti** [23]	55	F	–	L	+	ns	ns	RIC	–	12	HH, N/V	HR, Het L	+	Excision	Improvement
**2019 Hasegawa** [24]	7	F	ns	L	ns	GKS Twice	15,5^a^	–	+	5	HP	Het L	–	Excison	Improvement
**Case 1**	39	F	+	L	+	Linac	18	–	–	12	HH	HR, Het L, Multi Nod E	–	FU, Excision	Improvement
**Case 2**	42	F	–	L	+	Linac	18	RIC	+	11	AS	HR, Het L, Nod E	+	Partial Excision	Improvement
**Case 3**	36	M	–	L	+	Linac	18	RIC	+	12	AS	Nod E	+	Cyst evacuation, Excision	Improvement
**Case 4**	31	M	–	L	+	Linac	18	RIC	+	10	HH, SD	HR, Het L	+	Partial Excision	Improvement
**Case 5**	62	M	+	L	+	Linac	18	–	–	13	HP	HR, Het L, Multi Nod E	+	Steroids	Stable

*ns* not specified, *IC* Internal Capsule, *BG* Basal Ganglia, *L* Lobar, *C* Cerebellum, *Linac* Linear Accelerator, *GKS* Gamma Knife Radiosurgery, *RIC* Radiation induced changes, *RN* Radionecrosis, *HH* Headache, *N/V* Nausea/Vomiting, *Vis* Visual deficit, *SD* Sensory Deficit, *Memory* Memory deficit, *E* Epilepsy, *AS* Asymptomatic, *AT* Ataxia, *HR* Hypointense Rim, *Het L* Heterogeneous Lesion, *Hypo L* Hypointense Lesion, *Nod E* Nodular Enhancement

^a^ Average Marginal Dose

^b^ Radiological and/or Clinical Improvement

## Results

In the 37 cases analyzed, the mean age was 33,7 years (sd 15,3 years) with a 1:1.2 male to female predominance. The characteristics of these patients are depicted in Table 1. Forty four percent of AVMs had initially bled. The nidus was located in the cerebral lobes in 69%, in the basal ganglia in 27,5% and in the cerebellum in 1 (3,4%) patient. Previous embolizations had been performed in 39,3% of patients. Radiation was delivered by gamma knife radiosurgery in 74,3% cases while the rest were treated with a linear accelerator. Six cases were irradiated 2 times and 1 case three times. The marginal dose had a mean of 20,3 Grays, sd 3,1 Grays. Before the development of CEIHs, RICs or radionecrosis in the years post radiosurgery had been observed in 32,4% patients. Expanding intracerebral hematomas were discovered after a mean of 7,7 years (sd 3,7 years) post SRS. On T2-weight imaging, CEIHs manifested as heterogeneous lesions in 54,1%, low intensity lesion in 20,8%, had a hypointense rim in 54,1%, and had nodular or modular enhancement on T1-weighted contrast enhanced in 45,8% of cases. A cystic component coexisting with the CEIH was observed in 62,1% of patients. Symptoms ranged from headache (44,8%), hemiparesis (41,3%), nausea/vomiting (13,7%), asymptomatic (10,3%), paresthesia (6,8%), visual disturbances (6,8%), ataxia (3,4%), seizures (3,4%), memory disturbances (3,4%) and facial palsy (3,4%). Angiographic obliteration of the cerebral AVM had been achieved in 78,3% of CEIHs.

Data on the therapeutic management were available in 35 patients. Complete excision was performed in 10 cases and led to clinical and/or radiological improvement in 9 patients, while 1 remained stable. Three cases were treated with partial excision with good results. Four patients had only the cystic component treated (2 patients with Omaya reservoir placement and 2 patients with evacuation). However, this approach failed to provide clinical improvement in 3 patients who eventually underwent complete excision with good results.

Follow-up or medical management with steroids was attempted in 18 patients of whom 16 eventually underwent excision and 1 partial excision because of lesion enlargement or non-improving symptoms. In this group, improvement was reported in 9 patient, 1 patient with partial excision experienced worsening while 7 patients were lost to follow-up. Surgery was judged hazardous in one patient (patient 5 in the present series) who remained clinically stable at 8 years follow-up.

## Discussion

Chronic encapsulated intracerebral hematomas are a very rare complication of radiosurgery for cerebral AVM. The incidence in our cohort of treated patients was 1,8% of patients with brain AVM treated with SRS over a period of 17 years. Other series attest to the rarity of this complication with reported incidences ranging from 0,6 to 4% [1, 16, 24, 25]. However, the long latency period of CEIHs and the cessation of imaging controls once nidus obliteration has been documented may have led to the underestimation of the true prevalence [26]. Longer periods of imaging follow-up, at least years post radiosurgery have been suggested [19].

Histologically CEIHs are made of a thickened hematoma capsule with abundant microvasculature that can easily bleed when removed surgically. The hematoma itself is serous and is usually easily aspirated. The gross appearance is similar to chronic subdural hematoma. CEIHs may develop near vascular lesions such as AVMs, cavernous angiomas and venous angiomas. It is thought that CEIHs develop secondary to hemorrhagic episodes of the initial angiomatous lesion with its eventual “self-destruction” or thrombosis [4, 16, 27, 28]. In the case of post radiosurgery obliterated AVMs, it is thought that radiation-induced inflammation triggers neo-angiogenesis of fragile new vessels, breakdown of the blood-brain barrier, fluid exudation in the nearby brain, edema and potential cyst formation. Dense vascularization has been found in the capsule of CEIHs and it is thought that bleeding of these fragile vessels results in expansion of the capsule and further bleeding, a mechanism similar to chronic subdural hematoma [23, 29–31]. Neovascularization and hematoma expansion appear to be mediated by VEGF (Vascular Permeability Factor), a potent vascular endothelial cell mitogen that promotes neovascularization and vascular permeability [22] associated also to chronic subdural hematoma pathophysiology. Further studies are needed to elucidate the mechanisms of CEIHs post AVM radiosurgery.

On MR imaging, a common finding in all cases of CEIHs was extensive perilesional edema. Most cases demonstrated as low intensity or heterogeneous lesions on T2 weighted imaging with or without a hypodense rim. On contrast enhanced T1 weighted images there existed usually nodular or multinodular enhancement (Table 1). CEIHs were associated with cyst in 62,1% of cases pointing to a possible common pathophysiologic mechanism [3]. The latency time from radiosurgery to CEIHs diagnosis was 7,7 years sd 3,7 years. Symptoms, the most common being from headache (44,8%), hemiparesis (41,3%), nausea/vomiting (13,7%) were mostly related to the mass effect of the gradually growing CEIH and the surrounding edema.

Several risk factors have been explored for the development of CEIHs including age, sex, basal ganglia AVM location, irradiated nidus volume, the marginal or total dose, early RICs, repeat radiosurgery, nidus obliteration, pre-radiosurgery embolization, pre-radiosurgery surgery and prior hemorrhage with inconsistent results [16, 25, 26, 28, 32]. In the present review, the distributions of age, sex, location, marginal dose, nidus obliteration and pre-radiosurgery embolization did not differ from distributions seen in cohorts of AVMs treated by radiosurgery. However, the incidence of radiation induced changes in the years post radiosurgery was unusually high (32,4%). There was also a high percentage (18,9%) of cases which had received repeat radiosurgery. Further studies are needed to ascertain the risk factors and mechanisms of CEIHs that develop post SRS for AVM [14].

CEIHs often caused progressive neurological deficits due to mass effect. The most efficient treatment was complete excision that led to clinical and/or radiological improvement in cases. Partial treatment was less efficient and had to be complemented by complete excision in cases. Conservative management consisting of follow-up or steroid administration was unsuccessful in most cases and had to be complemented by total excision of the hematoma and the capsule to achieve good clinical outcome.

This study is susceptible to a number of biases inherent to any retrospective study and review like the small number of cases, selection bias and publication bias. The time CEIHs were detected was mostly based on the timing of symptom development and asymptomatic CEIHs may have been underreported. Larger studies are needed to further elucidate the pathophysiology, incidence and risk factors related to the development of CEIHs post cerebral AVM radiosurgery.

## Conclusion

CEIHs are a rare late complication that develop after SRS to treat cerebral AVMs. A potential risk factor is the appearance of radiation induced changes post SRS. CEHIs become usually symptomatic because of their mass effect and extensive surrounding edema. To manage these symptoms, CEIHs should ideally be evacuated with complete capsule removal. Partial capsule removal in the case of lesions in eloquent regions may be an alternative treatment.

## Data Availability

The datasets during and/or analysed during the current study are available from the corresponding author on reasonable request.

## Abbreviations

SRS: Stereotactic Radiosurgery
bAVM: Brain Arteriovenous Malformation
CEIH: Chronic Encapsulated Intracerebral Hematoma
RIC: Radiation-induced change
STROBE: Strengthening the Reporting of Observational studies in Epidemiology
VEGF: Vascular Permeability Factor
